# Complications of Percutaneous Nephrolithotomy: Experience From a Tertiary Care Center

**DOI:** 10.7759/cureus.69096

**Published:** 2024-09-10

**Authors:** Udham Singh, Vivek K Singh, Vishwajeet Singh, Alka Singh, Jigardeep Singh

**Affiliations:** 1 Urology, King George’s Medical University, Lucknow, IND; 2 Pathology, Sanjay Gandhi Postgraduate Institute of Medical Sciences (SGPGIMS), Lucknow, IND

**Keywords:** calculus, hematuria, pcnl, pelvicalyceal system, staghorn, stone burden, urolithiasis

## Abstract

Introduction

Renal stones are mineral concretions in the pelvicalyceal system. Their prevalence and recurrence are increasing globally. Percutaneous nephrolithotomy (PCNL) is a minimally invasive procedure for the removal of kidney stones. It is a safer technique offering the highest stone-free rates. However, a few complications may still occur. We aimed to evaluate our experiences of PCNL and classify the complications as intraoperative, early postoperative, and late postoperative events. We also aimed to find the predictors of complications in PCNL.

Methods

A single-center prospective observational study was conducted from June 2021 to October 2022 where all patients who were >18 years old with radiopaque calculus in the kidney and underwent PCNL were included. Statistical analysis was performed using the IBM Statistical Package for Social Sciences (SPSS) software (IBM SPSS Statistics, Armonk, NY). A p-value of <0.05 was considered significant.

Results

Two hundred one patients including 137 males and 64 females participated in the study. The overall rate of complications was 21.9%. Most of the patients (16.4%) experienced minor complications of Clavien grades 1 and 2. A Clavien grade of >3 included major complications and was noted in 5.5% of patients. No mortality was seen in the postoperative period. Female patients (p = 0.028), a stone burden of >3 cm (p = 0.003), stones in multiple calyces (p = 0.001), hydronephrosis (p = 0.001), history of recently treated urinary tract infection (UTI) (p < 0.001), longer operative time (>91 minutes) (p < 0.001), Guy’s stone scores (GSS) III and IV (p < 0.001), complex renal calculi (staghorn) (p = 0.002), and multiple punctures (p = 0.001) were associated with higher complication rates after PCNL.

Conclusion

Most PCNL-related complications are minor and resolve with conservative or minimally invasive management. However, there are certain complications that can limit the surgical outcome. The overall complication rate in the current study is similar to that reported in the literature. Bleeding was the most common intraoperative complication, whereas hematuria was the most common early postoperative complication. A stone burden of >3 cm, hydronephrosis, longer operative time, higher GSS, and multiple punctures can all affect the rate of complications.

## Introduction

Renal stones are mineral concretions in the kidney pelvicalyceal system [1]. The prevalence and recurrence rate of kidney stone disease are increasing worldwide [2]. The increasing trend is probably associated with modifications in lifestyle such as the absence of physical activity, changes in dietary habits [3,4], and global warming [5]. Urolithiasis nearly affects 12% of the entire population of the world at some point in time [6]. It can affect people of any age, race, and sex [5,7]. In India, ~12% of people are affected by urinary stones, of which 50% usually end up with end-stage renal disease [8]. Over the past 30 years, the management of symptomatic kidney stones in both pediatric and adult populations has evolved enormously, from open surgical lithotomy techniques to minimally invasive endourological approaches. Extracorporeal shock wave lithotripsy (ESWL), rigid or flexible retrograde ureteroscopic stone fragmentation and retrieval, and percutaneous nephrolithotomy (PCNL) are the three most common modalities used for the management of renal stones [1].

PCNL was established in 1970, as a minimally invasive procedure for the removal of kidney stones [9,10]. The European Association of Urology (EAU) recommends PCNL as the first line of management for stones of >2 cm, multiple stones, and stones involving inferior calyces [11]. Large kidney stones, including staghorn and complex stones, can be removed by PCNL [12]. Improvements in lithotripsy technology (pneumatic devices/ultrasound and holmium/yttrium-aluminum-garnet laser), instruments used (ureteroscopes and flexible pyeloscopes), and the experience of treating surgeons have all contributed to increased efficacy of PCNL [13]. PCNL, however, can still be associated with complications. The “Clinical Research Office of the Endourological Society” (CROES), a recent multicenter study, showed an overall complication rate of 20.5% [14,15]. The rate of complications as high as up to 83% has also been reported [16]. A specialized grading system for the assessment of PCNL-related complications is currently lacking. The most widely accepted reporting system used currently is the modified Clavien-Dindo classification for postoperative surgical complications, and most of the previous studies have described the postoperative complications of PCNL using this system. However, none of the classification systems proposed until now have become standard due to their inherent fallacies and also because of their inability to successfully classify the postoperative complications of PCNL [17].

So, we aimed to evaluate our experiences of PCNL and classify the complications noted as intraoperative, early postoperative, and late postoperative events. We also aimed to find out the risk factors for PCNL-associated complications.

## Materials and methods

A single-center prospective observational study was conducted in the Department of Urology at King George’s Medical University, Lucknow, from June 2021 to October 2022. All adult patients (age of >18 years) with large renal stones of >2 cm, partial/complete staghorn stones, and multiple renal stones were included in the study. The exclusion criteria were bleeding diathesis, patient on anticoagulant therapy, active urinary tract infection (UTI), high-risk patient for anesthesia due to cardiopulmonary involvement, and patients unwilling to undergo the PCNL. Informed consent was obtained from all the patients. All clinically relevant factors that can influence postoperative complications were included in the study. Clinical and demographic data were obtained from the patient’s medical record files. Ethical clearance for the study was obtained from the Institutional Ethics Committee of King George’s Medical University, Uttar Pradesh (UP). The approval number for the study is XI-PGTSC-IIA/P10.

Preoperative evaluation

All patients with renal calculi were subjected to further investigations: preoperative complete blood count (CBC), kidney function test (KFT), coagulation profile, urine analysis, and culture sensitivity. The radiological assessment of stone size, location, anatomy, and function of both kidneys was done through kidney, ureter, and bladder (KUB) X-ray, intravenous urography (IVU), ultrasonography (USG), non-contrast or contrast-enhanced computed tomography (NCCT/CECT), and renal scintigraphy (only when indicated). Cases with an existing urinary tract infection (UTI) were treated with an antibiotic course based on their urine culture and sensitivity report, before the procedure. After ensuring sterile urine, the patients underwent PCNL. Intravenous antibiotic prophylaxis was given to all patients before anesthesia. Patients with evidence of sepsis, pyonephrosis, or obstructive uropathy first underwent preoperative drainage by percutaneous nephrostomy (PCN) or double-J stent (DJS) placement, followed by PCNL after 2-4 weeks. None of the patients underwent bilateral simultaneous PCNL. Patients with bilateral renal calculi underwent surgery at a gap of four weeks. Stone burden calculated as the largest stone dimension was measured on preoperative radiography. Stone complexity was defined as simple-solitary and multiple or staghorn (partial/complete) calculi and was also defined using Guy’s stone score (GSS).

Surgical procedure

All PCNL procedures were performed by a team of experienced consultant urologists. The decision to proceed with PCNL under general anesthesia (GA) or spinal anesthesia (SA) is primarily based on the anesthesia team assessment. The procedure began with cystoscopy, followed by retrograde catheterization using a 4F/5F ureteric catheter in the patient’s lithotomy position under GA or SA. Patients were moved into the prone position, and percutaneous access to the renal calyceal system was established by bull’s eye technique under C-arm fluoroscopic guidance after obtaining an air or contrast pyelogram through the ureteric catheter. By aspirating urine from the needle, the proper puncture of calyces was confirmed. A hydrophilic guidewire was inserted through the needle and placed into the ureter or coiled in the pelvicalyceal system. The tract was dilated with Alken’s serial metallic dilator (Karl Storz, Tuttlingen, Germany) over the central guide rod up to 24-30 Fr, and an Amplatz sheath was placed. A 22 Fr rigid nephroscope (Karl Storz, Tuttlingen, Germany) was used to perform nephroscopy. The calculi were identified and fragmented using pneumatic lithoclast (Swiss LithoClast, EMS, Nyon, Switzerland). To access difficult areas, a nephroscope with the removal of the outer sheath was used. Intraoperative percutaneous calyceal irrigation (IPCI) was used to remove the smaller fragments of stone, avoiding additional puncture. The cases with staghorn or complex renal stones generally required multiple tract dilatation for complete stone clearance. The clearance of the stone was confirmed by nephroscopy and fluoroscopy before the termination of the procedure, and a double-J (DJ) stent was placed if needed. If the patient was planned for relook PCNL or if DJ stent placement was not done, an external ureteral catheter was left in situ. It was not considered a complication. DJ stent was removed after 2-4 weeks. Postoperative CBC and KFT were obtained for each patient. A plain KUB X-ray and USG were performed on the second postoperative day to confirm stone clearance. At our hospital, usually 48 hours after the PCNL procedure, we clamp the nephrostomy tube. If there are no fever and no pain, the nephrostomy tube is removed in uneventful cases. The following operative and postoperative factors were recorded: primary calyceal access, the number of tracts, the duration of surgery, stone-free status, drop in hemoglobin, intraoperative and postoperative complications, the total duration of hospital stay, and the rate of ancillary procedures.

The result of the procedure was classified as complete stone clearance or the presence of residual stones or the presence of clinically insignificant residual fragments (CIRF). CIRF was considered when the residual stone fragment was <4 mm, noninfectious, non-obstructing, and asymptomatic. The procedure was considered successful if the patient had either complete stone clearance or CIRF. Complications were recorded as intraoperative, early postoperative, and late postoperative events. Early postoperative (≤30 days) and late postoperative (>30 days) were used for reporting complications following PCNL. All patients were followed up at 2-4-week and 3-4-month intervals to see for any late postoperative complications. Ultrasonography, plain X-ray, CECT KUB, and diethylenetriaminepentaacetic acid (DTPA) renal scan were performed (if indicated). Serum creatinine and urine analysis were also performed. Complications were graded on the basis of the modified Clavien-Dindo classification. This classification was proposed for grading postoperative complications of general surgical procedures, which ranked the severity of surgical complications. Later on, the same classification system was used to classify the complications of urological procedures such as open/laparoscopic radical prostatectomy, live donor nephrectomy, pyeloplasty, partial/radical nephrectomy, and endourological procedures such as transurethral resection of the prostate (TURP). Mandal et al. reported the modified Clavien-Dindo classification for grading postoperative complications of PCNL [17].

Statistical analysis

Categorical variables were presented in number and percentage, and continuous variables were presented as mean and standard deviation (SD). Quantitative variables were compared using the Mann-Whitney U test/unpaired t-test as appropriate between the two groups. Qualitative variables were compared using the chi-square test/Fisher’s exact test as appropriate. In assessing risk factors for complications, binary logistic regression analysis was used. A p-value of <0.05 was considered statistically significant. The data was entered in an MS Excel (Microsoft Corp., Redmond, WA) spreadsheet, and analysis was done using Statistical Package for Social Sciences (SPSS) version 23.0 (IBM SPSS Statistics, Armonk, NY).

## Results

Two hundred one patients including 137 (68.2%) males and 64 (31.8%) females participated in the study. The mean patient age was 39 ± 14 years (range: 18-78 years). The majority (47.3%) of the patients were in the age group 18-35 years. The most common presenting complaint was flank pain (98.5%). Thirty-two (15.9%) patients had associated comorbidities, and 33 (16.4%) had a previous history of stone procedures. Four (2.0%) patients had congenital anomalies: horseshoe kidney (three, 1.5%) and right malrotated kidney (one, 0.5%). The demographic details of the study cohort have been displayed in Table 1.

**Table 1:** Demographic details of the study cohort. SD: standard deviation

Age (years) (mean ± SD)	39 ± 14	
Age intervals	Frequency (N)	Percentage (%)
18-35 years	95	47.3
36-50 years	60	29.9
51-65 years	38	18.9
>65 years	8	4.0
Gender		
Male	137	68.2
Female	64	31.8
Symptoms		
Flank pain	198	98.5
Hematuria	3	1.5
Associated comorbidities		
Coronary artery disease	2	1.0
Chronic kidney disease	7	3.5
Hypertension (HTN)	9	4.5
HTN and mitral stenosis	1	0.5
HTN and hypothyroidism	1	0.5
Type 2 diabetes mellitus (T2DM)	3	1.5
T2DM and HTN	9	4.5
Congenital anomalies		
Horseshoe kidney	3	1.5
Right malrotated kidney	1	0.5
History of previous stone procedure	33	16.4

Forty-six of 201 (22.9%) patients had positive preoperative urine cultures, and the most common organism isolated was *Escherichia coli* (32), followed by *Klebsiella* (seven) (Table 2).

**Table 2:** Laboratory findings of the study cohort. SD: standard deviation

Parameters		
Hemoglobin (g/dL)	Mean ± SD	
Preoperative hemoglobin	13.04 ± 1.75	
Postoperative hemoglobin	11.17 ± 1.70	
Serum creatinine (mg/dL)	Mean ± SD	
Preoperative serum creatinine	1.05 ± 0.57	
Postoperative serum creatinine	1.16 ± 0.63	
Urine culture	Frequency (N)	Percentage (%)
Escherichia coli	32	15.9
Enterococcus	4	2.0
Klebsiella	7	3.5
Pseudomonas	3	1.5

One hundred three (51.2%) patients had right renal calculus, while 82 (40.8%) had left renal calculus. Eleven (5.5%) had bilateral renal calculi, and five (2.5%) had upper ureteric calculus. Ninety (44.8%) patients had multiple renal calculi. The most common location of the stone was the renal pelvis (53.2%). The mean stone size was 21 ± 7 mm, and a stone burden of >3 cm was present in 24 (11.9%) patients. The most common Guy’s stone scores were I (47.3%) and II (38.8%). One hundred sixty-six (82.3%) patients had associated hydronephrosis, graded as mild, 109 (54.2%); moderate, 38 (18.9%); and severe 19 (9.5%), based on the Society of Fetal Urology (SFU) grading system for hydronephrosis on ultrasonography. Preoperative DJ stenting/PCN was performed in 27 (13.5%) patients. In 107 (53.2%) patients, the right PCNL was performed. The left PCNL was performed in 94 (46.8%) patients. The mean operative time was 79 ± 17 minutes. The procedure was performed under spinal anesthesia (190, 94.5%) or general anesthesia (11, 5.5%). The decision of taking the patient for PCNL under GA/SA was primarily based on the decision of the anesthesiologist. In most patients, an infra-costal puncture of the renal calyx was performed (150, 74.6%). Supra-costal puncture was performed in only 29 (14.4%) patients. Multiple punctures, including supra-costal and infra-costal punctures together, were performed in 22 (10.9%) patients, primarily for larger stone burden. Clinical details of the study cohort have been presented in Table 3.

**Table 3:** Clinical findings of the study cohort and stone characteristics. SD, standard deviation; DJ, double-J; PCN, percutaneous nephrostomy; PCNL, percutaneous nephrolithotomy

Mean stone size (mm) (mean ± SD)	21 ± 7	
Laterality	Frequency (N)	Percentage (%)
Right renal calculus	103	51.2
Left renal calculus	82	40.8
Bilateral renal calculi	11	5.5
Upper ureteric calculus	5	2.5
Multiplicity		
Single stone	111	55.2
Multiple stones	90	44.8
Location		
Renal pelvis	107	53.2
Inferior calyx	35	17.4
Middle calyx	17	8.5
Partial staghorn	12	6.0
Staghorn	11	5.5
Superior calyx	9	4.5
Middle and inferior calyx	5	2.5
Upper ureter	5	2.5
Stone burden (>3 cm)	24	11.9
Hydronephrosis		
Mild	109	54.2
Moderate	38	18.9
Gross/severe	19	9.5
Preoperative DJ stent/PCN	27	13.5
Procedure performed		
Right PCNL	107	46.8
Left PCNL	94	53.2
Anesthesia performed		
Spinal	190	94.5
General	11	5.5
Operative time (mean ± SD) (minutes)	79 ± 17	

In 169 (84.1%) patients, successful PCNL was achieved, where complete stone clearance was obtained in 163 (81.1%) patients and CIRF was noted in six (3%) patients. Overall residual kidney stones after the PCNL procedure were present in 32 (15.9%) patients, of whom relook PCNL was performed in 15 (7.5%) patients and ESWL in 17 (8.5%) patients. In 15 cases of relook PCNL, nine patients had complete clearance, and six patients had clinically significant residue of >4 mm at the end of the procedure. These six patients were offered ESWL, but they denied further procedures. The ESWL as an ancillary procedure was performed on 17 patients, of whom eight patients had complete clearance at the end of three months of follow-up. The remaining nine patients still had residual fragments, but they denied further treatment. The overall complete stone clearance rate (after ancillary procedures) was 92.5%. The mean duration of hospital stay was 4.6 ± 1.8 days. Stone clearance rates after PCNL monotherapy and ancillary procedure have been displayed in Table 4.

**Table 4:** Stone clearance after PCNL monotherapy and ancillary procedure. PCNL, percutaneous nephrolithotomy; ESWL, extracorporeal shock wave lithotripsy

Stone clearance	Frequency (N)	Percentage (%)
Complete	163	81.1
Clinically insignificant residual fragment (CIRF)	6	3.0
Residual calculus	32	15.9
Ancillary procedure for residual calculus		
Relook PCNL	15	7.5
ESWL	17	8.5
Final stone clearance after ancillary procedure		
Complete clearance	186	92.5
Residual calculus	15	7.5

**Figure 1:**
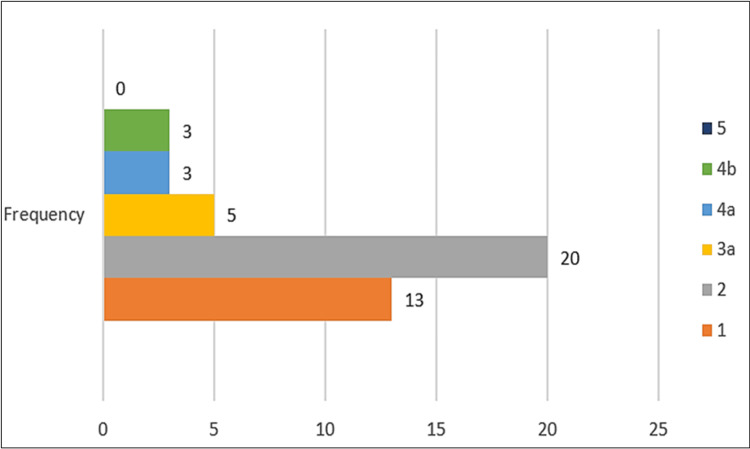
Modified Clavien-Dindo grading of postoperative complications following PCNL. PCNL: percutaneous nephrolithotomy

The overall rate of complications was 21.9%. Twenty-two (10.9%) patients developed intraoperative complications during PCNL, whereas 44 (21.9%) developed early postoperative complications after the procedure. All 22 patients who developed intraoperative complications also developed early postoperative complications. Most patients (33, 16.4%) experienced minor complications (Clavien grades 1 and 2). Clavien grades of ≥3, considered major complications, were noted in 11 (5.5%) patients (Figure 1).

**Figure 2:**
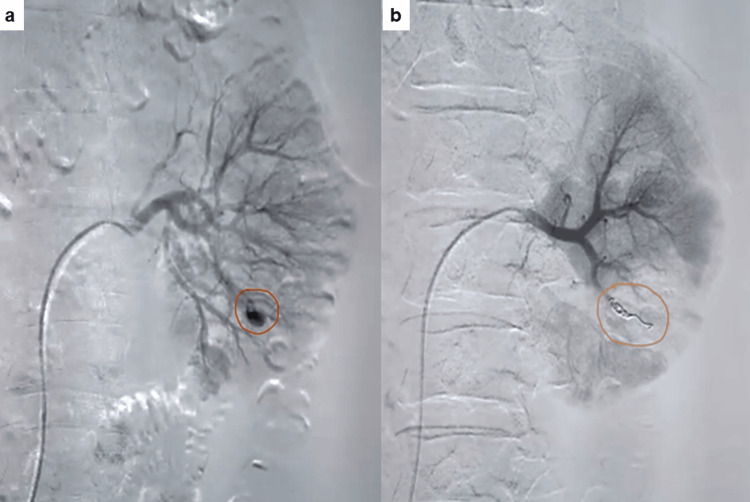
Post-PCNL digital subtraction angiography (DSA) showing (a) pseudoaneurysm on renal angiography and (b) management by super-selective coil embolization (highlighted by circles). PCNL: percutaneous nephrolithotomy

Among intraoperative non-urological complications, four (2.0%) patients developed hypotension and hypothermia; however, only one of them required intensive care unit (ICU) care. In one patient, the procedure had to be abandoned due to the sudden onset of arrhythmia intraoperatively. During the early postoperative period, 17 (8.4%) patients developed hypotension, probably due to the effect of spinal anesthesia. One patient developed myocardial ischemia (MI), and one developed arrhythmia; however, no one required ICU care. Among the intraoperative urological complications, bleeding was most common (20, 9.9%), followed by renal pelvis perforation (three, 1.5%). Because of severe bleeding, the procedure was abandoned in two (1%) patients. No patient developed pulmonary complications and adjacent organ injury or required a blood transfusion. Hematuria (37, 18.4%) was the most common complication in the early postoperative period, followed by fever (26, 12.9%). Twenty (9.9%) patients required blood transfusion following hematuria, and three (1.5%) required super-selective renal angioembolization for persistent hematuria (Figure 2).

**Figure 3:**
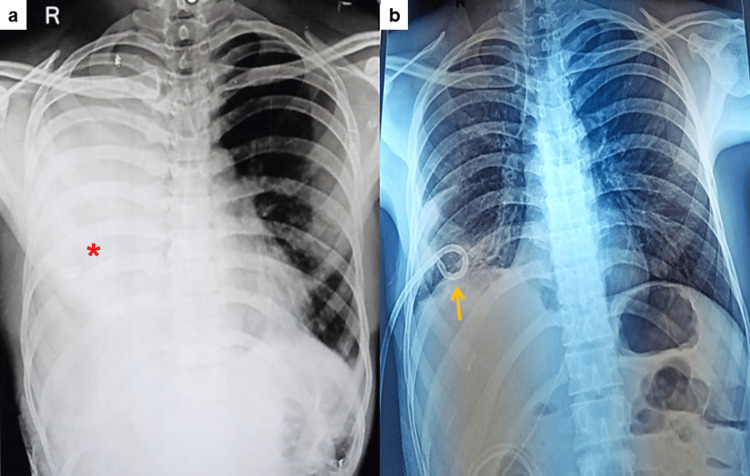
Post-PCNL chest X-ray (POD 3rd) showing (a) massive hydrothorax (highlighted by *) and (b) management with intercostal drainage (highlighted by arrow). PCNL, percutaneous nephrolithotomy; POD, postoperative day

Three (1.5%) patients developed hydrothorax, which was managed by intercostal drainage (ICD) (Figure 3). Hydrothorax is a well-known complication of PCNL, particularly if the initial puncture and dilatation of the tract are done above the 12th rib (supra-costal puncture). It can be associated with concomitant pneumothorax also.

None of the patients had other organ injury in the immediate postoperative period. In the present study, 10 patients developed a urinary tract infection (UTI) following PCNL. Among them, two patients had an infection with *Pseudomonas aeruginosa*, and one patient had a *Klebsiella* infection resistant to multiple drugs. These three patients (1.5%) developed urosepsis but were sensitive to meropenem. All received intravenous meropenem for five days along with other supportive measures. Their follow-up urine cultures were sterile. All patients were followed up for three months. None developed late postoperative complications such as arteriovenous fistula, the impairment of renal function, persistent urinary leakage, secondary pelviureteric junction obstruction (PUJO), or urethral stricture. Also, no mortality was seen in the postoperative period. The intraoperative and early postoperative complications have been displayed in Table 5.

**Table 5:** Intraoperative and early postoperative complications of PCNL. PCNL: percutaneous nephrolithotomy

Intraoperative non-urological complications due to general anesthesia	Frequency (N)	Percentage (%)
Hypotension	1	0.5
Hypothermia	1	0.5
Arrhythmia	1	0.5
Intraoperative non-urological complications due to spinal anesthesia		
Hypotension	3	1.5
Hypothermia	3	1.5
Intraoperative urological complications		
Bleeding	20	10.0
Renal pelvis perforation	3	1.5
Abandoned procedure due to bleeding	2	1.0
Early postoperative non-urological complications		
Hypotension	17	8.5
Myocardial ischemia	1	0.5
Arrhythmia	1	0.5
Early postoperative urological complications		
Hematuria	37	18.4
Fever	26	12.9
Blood transfusion	20	10.0
Urine leakage	15	7.5
Transient rise of serum creatinine	11	5.5
Urinary tract infection	10	5.0
Respiratory distress	7	3.5
Wound infection	6	3.0
Stent migration	2	1.0
Hydrothorax/pneumothorax	3	1.5
Angioembolization	3	1.5
Urosepsis	3	1.5
Readmission for hematuria	5	2.5

Patients with a stone burden of >3 cm (p = 0.003), stones in multiple calyces (p = 0.001), associated hydronephrosis (p = 0.001), and a history of recently treated UTI (p < 0.001) had a greater likelihood of developing complications. Female patients (31.2%) developed more complications than males (17.5%) (p = 0.028). Longer operative time (>91 minutes) (p < 0.001), GSS III and IV (p < 0.001), complex renal calculi (staghorn) (p = 0.002), and multiple punctures (p = 0.001) were also significantly associated with higher complication rates after PCNL. Risk factors for PCNL-associated complications have been displayed in Table 6 and Table 7.

**Table 6:** Risk factors for PCNL-associated complications. HSK, horseshoe kidney; PCNL, percutaneous nephrolithotomy

Preoperative variables (n = 201)		N	Complications		P-value
			Frequency	%	
Sex	Male	137	24	17.5	0.028
	Female	64	20	31.2	
Age intervals (years)	18-35	95	17	17.9	0.326
	36-50	60	14	23.3	
	51-65	38	12	31.6	
	Above 65	8	1	12.5	
Previous stone procedure	Yes	33	8	24.2	0.721
	No	168	36	21.4	
Associated comorbidity	Yes	32	5	15.6	0.894
	No	169	39	23.1	
Congenital anomalies (HSK and malrotation)	Yes	4	1	33.3	0.775
	No	197	43	21.8	
Stone burden (>3 cm)	Yes	24	11	45.8	0.003
	No	177	33	18.6	
Multiplicity	Single	111	15	13.5	0.001
	Multiple	90	29	32.2	
Hydronephrosis	Gross/severe	19	10	52.6	0.001
	Moderate	38	12	31.6	
	Mild	109	17	15.6	
	No	35	5	14.3	
Urine culture	Escherichia coli	32	7	21.9	<0.001
	Enterococcus	4	1	25.0	
	Klebsiella	7	5	71.4	
	Pseudomonas	3	3	100.0	
	Sterile	155	28	18.1	
Preoperative double-J (DJ) stent	Yes	19	7	36.8	0.098
	No	182	37	20.3	
Preoperative percutaneous nephrostomy (PCN)	Yes	8	3	37.5	0.376
	No	193	41	21.2	

**Table 7:** Risk factors for PCNL-associated complications. SD, standard deviation; PCNL, percutaneous nephrolithotomy; ASA, American Society of Anesthesiologists

Variables		N	Complications		P-value
			Frequency	%	
Guy’s stone score (GSS)	I	95	12	12.6	<0.001
	II	78	19	24.4	
	III	12	7	58.3	
	IV	11	6	54.5	
ASA grading	I	179	41	22.9	0.506
	II	19	3	15.8	
	III	3	0	0.0	
Supra-costal puncture	Yes	32	9	28.1	0.352
	No	169	35	20.7	
Infra-costal puncture	Yes	178	40	22.5	0.579
	No	23	4	17.4	
Multiple puncture	Yes	22	11	50.0	0.001
	No	179	33	18.4	
Location	Inferior calyx	35	5	14.3	0.002
	Middle and inferior calyx	5	2	40.0	
	Middle calyx	17	4	23.5	
	Renal pelvis	107	17	15.9	
	Superior calyx	9	3	33.3	
	Upper ureter	5	0	0	
	Partial staghorn	12	7	58.3	
	Staghorn	11	6	54.5	
	Complications				
Operative time (minutes) (mean ± SD)	Yes		No		<0.001
	91 ± 17		75 ±16		

In the multivariate binary logistic regression analysis, sex (female), stone burden (>3 cm), and multiplicity (multiple stones) were significant risk factors for complications associated with PCNL. Females had higher odds of complications (standard error {SE} = 0.387, OR = 2.902, 95% confidence interval {CI} = 1.360-6.194, and p = 0.006). The presence of stone burden (>3 cm) increased the odds (SE = 0.486, OR = 4.118, 95% CI = 1.588-10.684, and p = 0.004). The presence of multiple stones was also associated with higher odds of complications (SE = 0.385, OR = 3.692, 95% CI = 1.736-7.850, and p = 0.001). These are displayed in Table 8.

**Table 8:** Multivariate logistic regression analysis of risk factors of complications after percutaneous nephrolithotomy. SE, standard deviation; CI, confidence interval

Clinical parameters	SE	P-value	OR	95% CI	
				Lower	Upper
Sex (female)	0.387	0.006	2.902	1.360	6.194
Stone burden (>3 cm)	0.486	0.004	4.118	1.588	10.684
Multiplicity (multiple stones)	0.385	0.001	3.692	1.736	7.850

## Discussion

PCNL is the treatment of choice for larger kidney stones (>2 cm) and is associated with higher stone-free rates. It is a minimally invasive approach and is considered a safe technique [18,19]. However, PCNL may be associated with a few serious complications such as post-procedural hematuria, injuries to adjacent organs, and infections that can be life-threatening [20,21]. Most of these complications are generally minor and resolve by itself without the need for any additional treatment or intervention [15,16].

The overall rate of complications noted was 21.9%, which was very close to the observation made by a large prospective multicenter study on 5724 PCNL procedures (20.5%) [15]. A retrospective single-center review reported a complication rate of 18.3% [20]. Postoperative complications of PCNL stratified as Clavien grades 1 and 2 are considered minor, whereas Clavien grades of ≥3 are considered major complications [15]. However, terms such as minor and major have not been standardized yet. A large multicentric study performed on >1000 PCNL procedures also stratified the complications into minor and major categories. Minor complications were fever (21%-32.1%), need for blood transfusion (11.2%-17.5%), and urinary leakage (7.2%), whereas major complications were septicemia (0.3%-4.7%), colonic injury (0.2%-0.8%) and pleural injury (0.0%-3.1%) [16]. Kallidonis et al. in their review rated the frequency of major complications as 2.8%-32.1% for infection, 0%-20% (overall: 7%) for bleeding, 1.5%-4.6% for neurocutaneous fistula, <5.2% for pelvicalyceal rupture, <2% for pleural injury, and <0.5% for colonic injury [22]. Of the patients in our study, 16.4% had minor complications (Clavien grades 1 and 2), and only 5.5% had major complications (Clavien grade of ≥3). The major complications noted were urosepsis requiring management in the intensive care unit, three (1.5%); severe hematuria requiring super-selective renal angioembolization, three (1.5%); hydrothorax requiring ICD, three (1.5%); MI, one (0.5%); and arrhythmia, one (0.5%). Other major complications such as nephrocutaneous fistula and injuries to the colon, duodenum, spleen, and liver were not encountered in our study.

Xun et al. [23] and Xu et al. [24] demonstrated that female sex is an independent predictive factor for complications after PCNL. In our study, the complication rate in females was twice more compared to males (31.2% versus 17.5%). Perioperative risk evaluation is performed according to the American Society of Anesthesiologists (ASA) classification and is considered a predictor of postoperative outcome [25]. However, it is not specific for urological procedures, and neither is specific for the risk of postoperative complications. Patel et al. in their study identified that the overall rate of complications after PCNL was similar in both high-risk ASA (ASA III or IV) and low-risk ASA (ASA I or II) patients [21]. Our results showed similar findings, suggesting that ASA score alone is not a predictor of complications after PCNL surgery (p = 0.506).

Chen et al. observed that complex renal stones (GSS III and IV) are well-established independent predictive factors for complications in PCNL. This is attributed to longer operative time and the need for multiple dilation and punctures, including punctures in the upper pole of the kidney [26]. Falahatkar et al. reported multiple punctures as an independent predictive factor for complications in PCNL [27]. Twenty-two (10.9%) patients underwent multiple punctures in the present study, and the overall rate of complication was 50%, compared to 18.4% in cases with a single puncture. The treatment of complex renal stones (GSS III-IV) remains a challenge, and staged procedures may reduce the complication rates [27]. Our study suggests that the total duration of surgery is an important predictor for complications in PCNL. The complications were present more frequently in patients with longer mean operative time (>90 minutes). A study by Labate et al. reported similar findings [15].

The multivariate binary logistic regression analysis showed that factors such as female gender, multiple stones, and a stone burden of >3 cm are associated with increased odds of complications. Females had nearly three times higher odds, which may suggest underlying biological differences or risk factors. Multiple stones and a stone burden of >3 cm increased the odds by over three to four times, highlighting their clinical significance. A study by Xun et al. reported similar findings [23].

Mandal et al. reported that complications with grade 2 severity are most common after PCNL. In their study, bleeding requiring blood transfusion was the most common individual complication, followed by fever [17]. Our study showed similar results, where hematuria was the most common individual complication observed in 37 (18.4%) patients, of which 20 (10%) required blood transfusion. In the literature, the rate of blood transfusion after PCNL ranges from 5% to 18% [28-32]. Blood transfusion was done postoperatively when hemoglobin dropped below 9 g/dL (due to intraoperative blood loss and/or low preoperative hemoglobin). The procedure was abandoned in two patients because of severe bleeding leading to hemodynamic instability and poor visualization. Three (1.5%) patients underwent super-selective renal angioembolization to control persistent hematuria. In a study by Richstone et al., 1.2% of patients required angioembolization for post-PCNL hematuria [33].

Fever was the second most common complication, noted in 26 (12.9%) patients in our study. In the literature, the incidence of fever post PCNL ranges from 6.5% to 13% [34,35]. Common causes of fever post PCNL may include UTI, wound infection, urosepsis, or urinoma formation. Fever may also occur as a side effect of blood transfusion. Ten (5%) patients presented with UTI in our study. Said et al. also reported similar findings [36]. Wound infection occurred in six (3%) patients in our study. All these patients responded well to proper cleaning, the application of povidone-iodine, and the removal of the nephrostomy tube post-PCNL procedure. At our hospital usually 48 hours after the PCNL procedure, we clamp the nephrostomy tube, and if there is no fever and no pain, then the nephrostomy tube is removed.

Pleural injury (hydrothorax/pneumothorax) was observed in three (1.5%) patients. All these patients had supra-costal punctures, which constituted 9.4% of total supra-costal punctures in the study. Said et al. in their study reported pleural injury in 1.2% of patients [36].

In our study, stone clearance after PCNL monotherapy was complete in 163 (81.1%) and CIRF in six (3%). Residual calculus was seen in 32 (15.9%) patients. Relook PCNL was performed in 15 patients, and ESWL was performed in 17 patients for residual renal calculi. The final stone clearance rate after these ancillary procedures was 92.5%. Goyal et al. reported a stone clearance rate of 85.4% after PCNL monotherapy and a final stone clearance rate of 93.7% after the ancillary procedures for residual calculus [37].

PCNL in the anomalous kidneys is a safe and feasible procedure similar to the normally located kidney [38]. Similar results were noted in our study where no significant complications were noted in patients with anomalous kidneys. Also, PCNL under spinal anesthesia is as effective and safe as PCNL under general anesthesia [39]. We observed similar findings.

Previous studies on complications of PCNL focused only on the modified Clavien-Dindo classification system, which mainly classifies the postoperative complications of PCNL but not the intraoperative ones. Our study suggests that further analysis on a larger study cohort is needed to characterize the PCNL-related complications in terms of intraoperative and postoperative events. However, our study is limited by a smaller sample size and non-blinded study protocol.

## Conclusions

PCNL is a safe renal stone extraction procedure and can rarely induce significant complications. While the majority of complications are minor and resolve with conservative or minimally invasive management, certain complications can limit surgical outcomes. The overall complication rate in the current study is similar to that reported in the literature. A stone burden of >3 cm, stones in multiple calyces, hydronephrosis, history of recently treated UTI, longer operative time, higher Guy’s stone scores, complex renal calculi, and multiple punctures can all affect the rate of complications in PCNL.
